# Rapid and Accurate Detection of *Mycobacterium tuberculosis* in Sputum Samples by Cepheid Xpert MTB/RIF Assay—A Clinical Validation Study

**DOI:** 10.1371/journal.pone.0020458

**Published:** 2011-06-29

**Authors:** Andrea Rachow, Alimuddin Zumla, Norbert Heinrich, Gabriel Rojas-Ponce, Bariki Mtafya, Klaus Reither, Elias N. Ntinginya, Justin O'Grady, Jim Huggett, Keertan Dheda, Catharina Boehme, Mark Perkins, Elmar Saathoff, Michael Hoelscher

**Affiliations:** 1 Division of Infectious Diseases and Tropical Medicine, Medical Center of the University of Munich (LMU), Munich, Germany; 2 National Institute of Medical Research—Mbeya Medical Research Programme, Mbeya, Tanzania; 3 Department of Infection, Windeyer Institute of Medical Sciences, University College London Medical School, London, United Kingdom; 4 Swiss Tropical and Public Health Institute (Swiss TPH), Basel, Switzerland; 5 Foundation for Innovative New Diagnostics (FIND), Geneva, Switzerland; New York State Health Department and University at Albany, United States of America

## Abstract

**Background:**

A crucial impediment to global tuberculosis control is the lack of an accurate, rapid diagnostic test for detection of patients with active TB. A new, rapid diagnostic method, (Cepheid) Xpert MTB/RIF Assay, is an automated sample preparation and real-time PCR instrument, which was shown to have good potential as an alternative to current reference standard sputum microscopy and culture.

**Methods:**

We performed a clinical validation study on diagnostic accuracy of the Xpert MTB/RIF Assay in a TB and HIV endemic setting. Sputum samples from 292 consecutively enrolled adults from Mbeya, Tanzania, with suspected TB were subject to analysis by the Xpert MTB/RIF Assay. The diagnostic performance of Xpert MTB/RIF Assay was compared to standard sputum smear microscopy and culture. Confirmed *Mycobacterium tuberculosis* in a positive culture was used as a reference standard for TB diagnosis.

**Results:**

Xpert MTB/RIF Assay achieved 88.4% (95%CI = 78.4% to 94.9%) sensitivity among patients with a positive culture and 99% (95%CI = 94.7% to 100.0%) specificity in patients who had no TB. HIV status did not affect test performance in 172 HIV-infected patients (58.9% of all participants). Seven additional cases (9.1% of 77) were detected by Xpert MTB/RIF Assay among the group of patients with clinical TB who were culture negative. Within 45 sputum samples which grew non-tuberculous mycobacteria the assay's specificity was 97.8% (95%CI = 88.2% to 99.9%).

**Conclusions:**

The Xpert MTB/RIF Assay is a highly sensitive, specific and rapid method for diagnosing TB which has potential to complement the current reference standard of TB diagnostics and increase its overall sensitivity. Its usefulness in detecting sputum smear and culture negative patients needs further study. Further evaluation in high burden TB and HIV areas under programmatic health care settings to ascertain applicability, cost-effectiveness, robustness and local acceptance are required.

## Introduction

Tuberculosis (TB) continues to kill 1.8 million people annually. Progress towards global TB control has remained elusive despite intensified standard measures of TB control [1]. National TB control programmes in most TB and TB/HIV endemic countries continue to rely largely on century old, antiquated and inaccurate tools such as direct smear microscopy, solid culture, chest radiography and tuberculin skin testing [2]. Currently, there is no rapid, point-of-care test that allows early detection of active TB at the peripheral health clinic level. Thus many patients with active TB, especially in TB and HIV endemic areas are either treated based on clinical grounds and without microbiological proof of TB-infection or remain undiagnosed and are continuing to spread the disease in the community [3]. The need for a more accurate, point-of-care TB diagnostic test that is applicable in TB and HIV endemic areas is crucial for achieving global TB control. Modeling studies show that new diagnostics for TB and multi-drug resistant TB (MDR-TB) may have an important impact at the population level in disease endemic countries [4]. Over the past decade the TB diagnostics pipeline has expanded, with several technologies showing promise [2]. Among them are new and simplified PCR-based methods which have been shown to detect *Mycobacterium tuberculosis* (*Mtb*) and resistance to rifampicin with good sensitivity and specificity directly from positive cultures or clinical specimens. Current large-scale roll-out of liquid culture promises to improve sensitivity and drug susceptibility testing (DST). However, caution should be applied in settings where non-tuberculous mycobacteria (NTM) are common environmental microorganisms, such as in sub-Saharan Africa [5], [6] because detection of NTM growth could lead to a false diagnosis of TB.

The World Health Organization (WHO) is ensuring that new TB diagnostic policies are evidence-based [7], and in alignment with the GRADE approach to guideline development [8]. In 2009, the first technical data were announced from an automated molecular test for TB, the Xpert MTB/RIF Assay co-developed by the Foundation for Innovative New Diagnostics, Cepheid (Sunnyvale, Calif, USA), and the University of Medicine and Dentistry of New Jersey [9]. This assay, which was CE (Conformité Européenne) marked in 2009, avoids many of the pitfalls of conventional nucleic acid amplification tests and can be performed by staff with minimal training, and used for case detection or MDR screening at the same time. Thus this assay is said to specifically detect *Mtb* and rifampicin resistance from one sputum sample within 2 hours. It is said to constitute a greatly simplified nucleic acid amplification test which can be performed by any laboratory or clinical personnel after minimal introductive training [9]. The Xpert MTB/RIF Assay is a single-use sample processing cartridge system that holds all required sample preparation and real-time PCR reagents and hosts the whole PCR reaction. The assay has recently undergone performance evaluation on respiratory specimens [9], [10], [11], [12] as well as on non-respiratory samples [11] [13], one of them was a multi-country ‘technical’ evaluation which showed the Xpert MTB/RIF Assay to have high sensitivity and specificity [10], producing results in 2 hours. Since December 2010 WHO recommends the use of Xpert MTB/RIF Assay as initial diagnostic test for TB diagnosis in patients with suspected MDR-TB or TB in association with HIV- infection [14]. In this cohort- study we wanted to validate the diagnostic accuracy of the Xpert MTB/RIF Assay in our setting, an area with high prevalence of TB and HIV. We compared the results of the Xpert MTB/RIF Assay to sputum smear and culture as standard diagnostic methods on samples from HIV-infected and HIV-uninfected patients with suspected TB, from Mbeya, Tanzania.

## Methods

### Ethics approval

The study was approved by the local Mbeya Medical Research and Ethics Committee and the National Institute of Medical Research (NIMR) Ethics Committee. Written informed consent was obtained from all participants or their legal representative for use of their sputum for TB diagnostics research.

### Study setting

The study was conducted according to the principles expressed in the Declaration of Helsinki as revised in 2000 at the Mbeya Referral Hospital in the Mbeya region, south west Tanzania which has a high burden of TB and HIV.

### Recruitment of participants

Three hundred consecutive patients with symptoms suggestive of pulmonary TB who presented to the Mbeya Referral Hospital between July 2007 and September 2007 were interviewed for enrolment into the study. After informed consent, recruitment procedures comprised interviews regarding medical history, clinical examination, chest radiography, blood sample collection and HIV pre- and post-test counseling of participants. Eight patients were excluded from the study because they either were incapable to produce sputum at recruitment or were lost to follow up during recruitment procedures.

### Sputum samples collected per patient

All 292 study patients had three sputum samples collected (one spot sample, one morning sample and another spot sample, making a total of three for analyses).

### Sputum sample processing

Sputum samples were split into two aliquots, one of which was stored at −80°C. The other aliquot was processed for standard sputum microscopy after Ziehl-Neelsen staining and culture. Sputa were decontaminated by the NALC-NaOH method and the resulting pellet was examined for acid fast bacilli (AFB) by microscopy and culture, always both on Lowenstein-Jensen media (LJ) and BACTEC MGIT (MGIT) 960 liquid culture (BACTEC™ MGIT, Becton Dickinson, Sparks, USA) in parallel, using standard protocols. The AFB smear grade was defined following the International Union against Tuberculosis and Lung Disease scale: Scanty (1–9/100 fields), 1+(10–99/100 fields), 2+(1–10/100 fields) and 3+(>10/field) [15]. A patient was considered smear positive if at least one smear was graded scanty or higher. Species determination of cultured organisms was performed by Genotype® Mycobacterium MTBC, CM and AS tests (Hain Lifescience, Nehren, Germany). Rifampicin susceptibility was tested using SIRE test kits in the BACTEC MGIT system [16].

The microbiology and the molecular biology laboratory of NIMR-Mbeya Medical Research Programme operate in accordance with standardized protocols and quality control and quality assurance procedures.

### Patient classification

Participants were classified into 5 groups according to their microbiological, radiological and clinical findings at enrollment and follow up visits:

#### TB (S+/C+)

Patients with microbiologically confirmed pulmonary TB who had at least one positive sputum-smear (S+) and one *Mtb*-positive sputum culture (C+) sample.

#### TB (S−/C+)

Patients with microbiologically confirmed pulmonary TB who were smear-negative but had at least one *Mtb*- positive culture sample.

#### No TB (C−)

Patients with bacterial chest infection who were sputum smear- and culture-negative and responded to antibiotic treatment with amoxicillin, co-trimoxazole, or cefpodoxime with full recovery. No anti-TB treatment was given to these patients.

#### Clinical TB (C−)

Patients who were classified as having pulmonary TB (despite lacking microbiological confirmation of *Mtb*) based on clinical and radiologic findings after failure to respond to two courses of oral antibiotics comprising amoxicillin, co-trimoxazole, or cefpodoxime. All patients showed a clear clinical response to TB treatment in the follow-up.

#### Indeterminate

Patients, who were lost to clinical follow up or had no complete set of data (either culture results or radiological data were unavailable). No anti-TB treatment was accorded.

### Patient treatment and follow up

All 292 patients were followed up for a period of 56 days. TB drug treatment was accorded by the District Leprosy and TB Coordinator following Tanzanian national guidelines for TB treatment. Patients diagnosed with HIV infection were referred for further staging and treatment to the relevant Care and Treatment Center.

### Analysis of sputum samples by Xpert MTB/RIF Assay

Biobanked sputum samples from 292 patients of all study groups were subject to analysis using Xpert MTB/RIF Assay during the period January 2010 to March 2010 at the same TB laboratory at MMRP. Clinical and laboratory staff involved with this study were blinded for clinical diagnosis, sputum smear, culture and drug susceptibility results of the sputa samples. Frozen sputa were thawed and processed according to the Xpert MTB/RIF Assay test procedure [9]. Positive results were displayed by Xpert MTB/RIF Assay as semi-quantitative estimates depending on the Ct value of the present sample. Lower cycle threshold (Ct) values represented a higher concentration of *Mtb* complex (MTBC) DNA and higher Ct values represented a lower DNA-concentration. A Ct value of ≥40 reflected a *Mtb-* negative sample.

### Statistical analysis

For statistical comparison, *Mtb* culture positivity was used as reference standard, defined as identification of *Mtb* in at least one positive standard sputum culture (either on LJ slopes or MGIT culture) out of three analysed sputum samples. Statistical analysis by number or type of sputum samples used by test performed was run using Stata 11.0 statistics software (Statacorp, College Station, TX, USA) [17]. Sensitivity and specificity were calculated using Stata's “diagt” component. Associations of indicators of AFB density between Xpert MTB/RIF Assay, sputum smear microscopy and culture results were assessed using linear regression and displayed using Stata graphics.

## Results

### Time to result and technical aspects

All sample results were obtained within two hours of commencing the analyses. There were no technical operational problems encountered with the use of the machine that required attention.

### Classification of the study population

A total of 292 individuals (151 females and 141 males) with a mean age of 39.2 years (SD = 13.8) were classified into five different groups according to microbiological, radiological and clinical findings (table 1).

**Table 1:** Patient classification, HIV prevalence and Xpert MTB/RIF Assay results for each patient group.

				Xpert MTB/RIF Assay result^a^	
		Size of group	HIV prevalence	positive	negative
Status	Group and definition	% of total (n)	% in group (n)	% in group (n)	% in group (n)
Microbiologically confirmed TB	**TB (S+/C+)**	17.5 (51)	66.7 (34)	98.0^b^ (50)	2.0 (1)
	**TB (S−/C+)**	6.2 (18)	88.9 (16)	61.1^c^ (11)	38.9 (7)
No microbiological proof of TB	**Clinical TB (C−)**	26.4 (77)	68.8 (53)	9.1 (7)	90.9 (70)
	**No TB (C−)**	35.3 (103)	48.5 (50)	1.0 (1)	99.0^d^ (102)
Incomplete clinical or microbiological assessment	**Indeterminate**	14.7 (43)	44.2 (19)	2.3 (1)	97.7 (42)
	**Total**	100 (292)	58.9 (172)	24.0 (70)	76.0 (222)

a = Xpert MTB/RIF Assay result per patient analysis.

b = sensitivity in this group, 95%CI = 89.6% to 100.0%.

c = sensitivity in this group, 95%CI = 35.7% to 82.7%.

d = specificity in this group, 95%CI = 94.7% to 100.0%.

S+/C+ = sputum smear positive and culture positive; S−/C+ = sputum smear negative and culture positive; C− = culture negative.

69 (23.6% of 292) patients had microbiologically confirmed pulmonary TB with *Mtb* identified in their sputum culture. These included 51 sputum smear-positive (S+/C+) and 18 smear-negative (S−/C+) cases. 103 patients (35.3% of 292) were shown to have no TB (C−). These patients were smear and culture negative and showed a sustained recovery within 56 days of receiving antibacterial antibiotic therapy. Out of the remaining 120 patients, 77 (26.4% of 292) were classified as clinical TB-cases (C−) on clinical and radiological grounds although sputum culture results were negative for *Mtb*. All patients in this group received anti- TB treatment and showed a sustained recovery after 56 days. Two study participants were smear-positive but culture- negative. These patients were included into this group due to typical clinical and radiological findings as well as marked improvement under anti −TB therapy. 43 participants (14.7% of 292) were classified as indeterminate and not included in the final analysis since there was no clear evidence of mycobacterial or other bacterial infection in those patients or sufficient data were not available for classification into one of the other groups.

### HIV status of participants

The overall HIV prevalence in the study population was 58.9% (95%CI = 45% to 89.9%). In microbiologically confirmed TB cases the HIV prevalence (66.7%) in group (S+/C+) was not statistically different (p-value 0.07) from HIV prevalence (88.9%) in group (S−/C+).

### Diagnostic performance analysis of the Xpert MTB/RIF Assay per classification group

In table 1 the results of Xpert MTB/RIF Assay as per patient analysis are depicted according to the patient classification group. 50 out of 51 smear and culture positive patients (S+/C+) were detected positive by Xpert MTB/RIF assay resulting in a 98% sensitivity (98% CI = 89.6% to 100.0%) in this group. Only 11 out of 18 patients with a negative smear and positive culture (S−/C+) were positive by the assay. Sensitivity was 61.1% (CI = 35.7% to 82.7%) in this group which is statistically different (p-value<0.001) to the sensitivity of the assay (98%) in group (S+/C+). Among all 103 TB-negative patients (C−) the submitted sputa of one patient were Xpert MTB/RIF Assay positive, giving the assay a specificity of 99% ( 95%CI = 94.7% to 100.0%). The corresponding patient was HIV-infected and had a previous history of TB and of receiving anti- TB treatment.

Amongst the 77 participants in the group of Clinical TB (C−) who had no *Mtb*- positive sputum culture but a clinical diagnosis of TB, the Xpert MTB/RIF Assay detected an additional seven (9.1% of 77) *Mtb-* positive patient samples. The Ct-values displayed by GeneXpert for these samples were high (Ct 22–28) or very high (Ct>28), indicating a low mycobacterial load in the sputum. Despite the low amplification signal, Xpert MTB/RIF Assay was consistently positive in the analysis of single or multiple sputum samples from these seven patients. All seven patients were HIV-positive and were thus treated for TB based on their clinical and radiological findings and non-responsiveness to anti-bacterial antibiotic therapy. None of them had a history of previous TB diagnosis or treatment. The sputum samples of the remaining 70 (90.9% of 77) participants of this group were Xpert MTB/RIF Assay negative.

### Diagnostic performance of the Xpert MTB/RIF Assay in per sample analysis and compared to other tests

For all 69 culture positive TB patients (S+/C+ and S−/C+), and 103 patients defined as TB negative (No TB [C−]), the diagnostic performance per sample (first spot sample and morning sample) of all the diagnostic tests alone and their combination were ascertained; the data is presented in table 2. The reference standard, on which microbiological confirmed TB diagnosis was based in this study, was defined as at least one positive sputum culture for *Mtb* out of three sputum samples analysed. Compared to spot sputa the sensitivity of the Xpert MTB/RIF Assay was only slightly increased in morning sputa showing no statistical difference (p-value 0.459); 84.1% (95%CI = 73.3% to 91.8%) vs. 88.4% (95%CI = 78.4% to 94.9%). Importantly, the overall per patient sensitivity of 88.4% (95%CI = 78.4% to 94.9%) of the Xpert MTB/RIF Assay remained the same when three sputa (first spot sample, morning sample and 2^nd^ spot sample) were analysed. In both types of sputa sensitivity of Xpert MTB/RIF Assay was always higher than that of any other method alone.

**Table 2:** Comparison of Xpert MTB/RIF Assay and other methods with reference standard for both HIV-positive and -negative participants.

Samples	Test^a^	Sensitivity (95%CI); n positive							Specificity (95%CI); n negative						
**Reference Standard** b								**69**							**103**
**One spot sputum**															
	Xpert	84.1	(	73.3	to	91.8	);	58	99.0	(	94.7	to	100	);	102
	Smear only	58.0	(	45.5	to	69.8	);	40	100.0	(	96.5	to	100	);	103
	LJ only	73.9	(	61.9	to	83.8	);	51	100.0	(	96.5	to	100	);	103
	Mgit only	76.8	(	65.1	to	86.1	);	53	100.0	(	96.5	to	100	);	103
	Smear & LJ	84.1	(	73.3	to	91.8	);	58	100.0	(	96.5	to	100	);	103
	Smear, LJ & Mgit	91.3	(	82.0	to	96.7	);	63	100.0	(	96.5	to	100	);	103
**One morning sputum**															
	Xpert	88.4	(	78.4	to	94.9	);	61	99.0	(	94.7	to	100	);	102
	Smear only	66.7	(	54.3	to	77.6	);	46	100.0	(	96.5	to	100	);	103
	LJ only	68.1	(	55.8	to	78.8	);	47	100.0	(	96.5	to	100	);	103
	Mgit only	78.3	(	66.7	to	87.3	);	54	100.0	(	96.5	to	100	);	103
	Smear & LJ	79.7	(	68.3	to	88.4	);	55	100.0	(	96.5	to	100	);	103
	Smear, LJ & Mgit	85.5	(	75.0	to	92.8	);	59	100.0	(	96.5	to	100	);	103
**First two sputa**															
	Xpert	88,4	(	78.4	to	94.9	);	61	99.0	(	94.7	to	100	);	102
	Smear only	71,0	(	58.8	to	81.3	);	49	100.0	(	96.5	to	100	);	103
	LJ only	87,0	(	76.7	to	93.9	);	60	100.0	(	96.5	to	100	);	103
	Mgit only	88,4	(	78.4	to	94.9	);	61	100.0	(	96.5	to	100	);	103
	Smear & LJ	89,9	(	80.2	to	95.8	);	62	100.0	(	96.5	to	100	);	103
	Smear, LJ & Mgit	95,7	(	87.8	to	99.1	);	66	100.0	(	96.5	to	100	);	103
**Per patient analysis (3 sputa)**															
	Xpert	88.4	(	78.4	to	94.9	);	61	99.0	(	94.7	to	100	);	102
	Smear only	73.9	(	61.9	to	83.8	);	51	100.0	(	96.5	to	100	);	103
	LJ only	94.2	(	85.8	to	98.4	);	65	100.0	(	96.5	to	100	);	103
	Mgit only	95.7	(	87.8	to	99.1	);	66	100.0	(	96.5	to	100	);	103
	Smear & LJ	95.7	(	87.8	to	99.1	);	66	100.0	(	96.5	to	100	);	103
	Smear, LJ & Mgit	100.0	(	94.8	to	100	);	69	100.0	(	96.5	to	100	);	103

a all culture results include speciation test results (for confirmation of M*tb* or exclusion of M*tb* in case when NTM present).

b reference standard for confirmed TB- diagnosis, defined as at least one positive culture (LJ or Mgit) confirmed as *Mtb* in speciation of per patient analysis; *Mtb-* negative defined as all cultures negative (LJ and Mgit) for *Mtb* in per patient analysis, speciation results included.

Smear = Sputum smear microscopy after ZN-staining; LJ = Loewenstein-Jensen culture on solid media; Mgit = BACTEC MGIT 960 liquid culture.

Specificity of Xpert MTB/RIF Assay was the same in spot and morning sputum (99%, 95%CI = 94.7% to 100.0%).

For analysis of 50 HIV-infected patients with a positive TB culture (S+/C+ and S−/C+) and 50 HIV-infected patients from the No TB (C−) group, the diagnostic results of the same tests as above are shown in table 3. In spot sputum the Xpert MTB/RIF Assay had 82% (95%CI = 68.6% to 91.4%) sensitivity compared to 88% (95%CI = 75.7% to 95.5%) sensitivity in morning sputum which was statistically not different (p-value 0.864). In both types of sputa specificity was 98% (95%CI = 89.4% to 100.0%).

**Table 3:** Comparison of Xpert MTB/RIF Assay and other methods with reference standard in HIV-positives only.

Samples	Test^a^	Sensitivity (95%CI); n positive							Specificity (95%CI); n negative						
**Reference Standard** b								**50**							**50**
**One spot sputum**															
	Xpert	82.0	(	68.6	to	91.4	);	41	98.0	(	89.4	to	100	);	49
	Smear only	52.0	(	37.4	to	66.3	);	26	100.0	(	92.9	to	100	);	50
	LJ only	68.0	(	53.3	to	80.5	);	34	100.0	(	92.9	to	100	);	50
	Mgit only	76.0	(	61.8	to	86.9	);	38	100.0	(	92.9	to	100	);	50
	Smear & LJ	78.0	(	64.0	to	88.5	);	39	100.0	(	92.9	to	100	);	50
	Smear, LJ & Mgit	88.0	(	75.7	to	95.5	);	44	100.0	(	92.9	to	100	);	50
**One morning sputum**															
	Xpert	88.0	(	75.7	to	95.5	);	44	98.0	(	89.4	to	100	);	49
	Smear only	60.0	(	45.2	to	73.6	);	30	100.0	(	92.9	to	100	);	50
	LJ only	68.0	(	53.3	to	80.5	);	34	100.0	(	92.9	to	100	);	50
	Mgit only	74.0	(	59.7	to	85.4	);	37	100.0	(	92.9	to	100	);	50
	Smear & LJ	76.0	(	61.8	to	86.9	);	38	100.0	(	92.9	to	100	);	50
	Smear, LJ & Mgit	84.0	(	70.9	to	92.8	);	42	100.0	(	92.9	to	100	);	50
**First two sputa**															
	Xpert	88,0	(	75,7	to	95,5	);	44	98,0	(	89,4	to	100	);	49
	Smear only	66,0	(	51,2	to	78,8	);	33	100,0	(	92,9	to	100	);	50
	LJ only	84,0	(	70,9	to	92,8	);	42	100,0	(	92,9	to	100	);	50
	Mgit only	86,0	(	73,3	to	94,2	);	43	100,0	(	92,9	to	100	);	50
	Smear & LJ	86,0	(	73,3	to	94,2	);	43	100,0	(	92,9	to	100	);	50
	Smear, LJ & Mgit	94,0	(	83,5	to	98,8	);	47	100,0	(	92,9	to	100	);	50
**Per patient analysis (3 sputa)**															
	Xpert	88.0	(	75.7	to	95.5	);	44	98.0	(	89.4	to	100	);	49
	Smear only	68.0	(	53.3	to	80.5	);	34	100.0	(	92.9	to	100	);	50
	LJ only	92.0	(	80.8	to	97.8	);	46	100.0	(	92.9	to	100	);	50
	Mgit only	96.0	(	86.3	to	99.5	);	48	100.0	(	92.9	to	100	);	50
	Smear & LJ	94.0	(	83.5	to	98.8	);	47	100.0	(	92.9	to	100	);	50
	Smear, LJ & Mgit	100.0	(	92.9	to	100	);	50	100.0	(	92.9	to	100	);	50

a all culture results include speciation test results (for confirmation of M*tb* or exclusion of M*tb* in case when NTM present).

b reference standard for confirmed TB- diagnosis, defined as at least one positive culture (LJ or Mgit) confirmed as *Mtb* in speciation of per patient analysis; *Mtb-* negative defined as all cultures negative (LJ and Mgit) for *Mtb* in per patient analysis, speciation results included.

Smear = Sputum smear microscopy after ZN-staining; LJ = Loewenstein-Jensen culture on solid media; Mgit = BACTEC MGIT 960 liquid culture.

### Comparison of quantitative results

**Figure 1:**
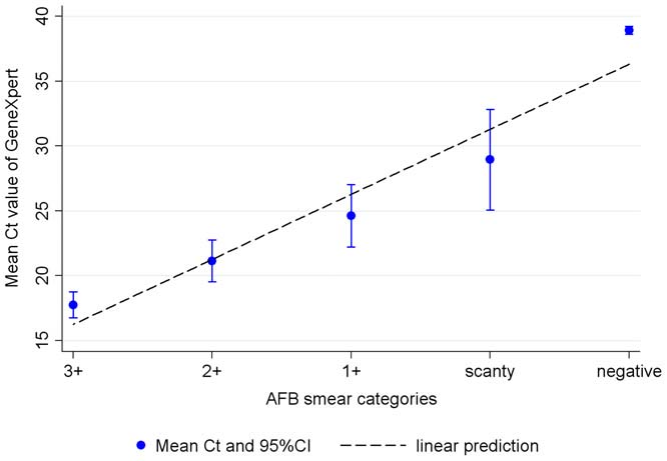
Cycle threshold (Ct-value) versus grade of smear-positivity. Ct versus degree of smear positivity, coded as negative = 0, scanty = 1, 1+ = 2 etc (regression coefficient β = −5.02, 95%CI = −7.44 to −2.60). Ct for Xpert MTB/RIF Assay negatives was coded as 40.

**Figure 2:**
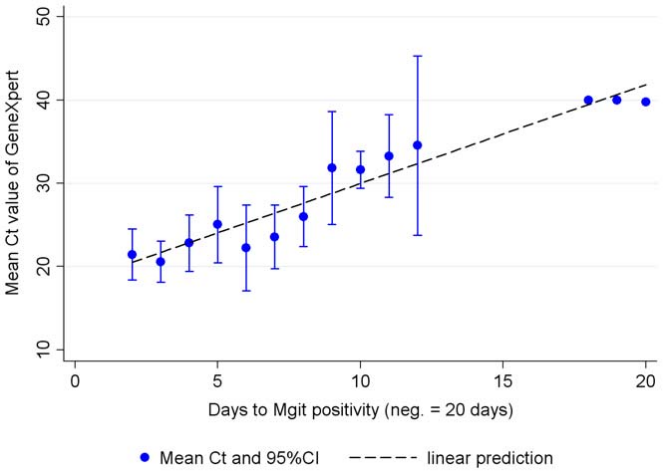
Cycle threshold (Ct-value) versus time to positivity in MGIT liquid culture. Ct versus days from inoculation into MGIT liquid culture to culture positivity as reported by the MGIT instrument (β = 1.19, 95%CI = 0.98 to 1.39). Ct for Xpert MTB/RIF Assay negatives was coded as 40.

An analysis of markers for sputum bacterial load, such as grade of smear- positivity (figure 1) and time to positivity in MGIT liquid culture (figure 2), shows good correlation with the quantitative result of real-time PCR obtained from the Xpert MTB/RIF Assay (expressed in Cycle threshold (Ct) values - which correlate inversely with the concentration of target DNA) (figure 1, 2).

### Specificity of Xpert MTB/RIF Assay in NTM - positive specimens

There were 45 patients (15.4% of 292), 24 in the No-TB (C−) group and 21 in the group of Clinical TB (C−), in whose cultures grew NTM. The speciation tests of these NTM detected M. fortuitum (10), M. intracellulare (5), M. celatum I+II (2), M. scrofulaceum (1) and M. szulgai (1). In 32 cases a final identification of the NTM was not possible. Only one of these patients' sputum samples tested positive by Xpert MTB/RIF Assay, resulting in an Xpert MTB/RIF Assay specificity of 97.8% in this group (95%CI = 88.2% to 99.9%).

### Drug resistance data on *Mtb* isolates

All strains of *Mtb* isolated were drug sensitive. There were no *Mtb* strains resistant to RIF or INH when tested by standard drug sensitivity testing (DST) methods on liquid culture. There was also no RIF resistance detected in any of the sputum samples processed by the Xpert MTB/RIF Assay.

## Discussion

Our study has several important and novel findings: Firstly, this is a relatively large, controlled, comprehensive clinical validation study of the diagnostic accuracy of the Xpert MTB/RIF assay in a clinical cohort of patients suspected of having active TB from a TB and HIV endemic area. More than 58% of the included subjects were HIV-infected. Secondly, we present data showing that Xpert MTB/RIF Assay has a very high sensitivity and specificity when tested in the field compared to the current reference standard. Thirdly, our data suggests that HIV status of patients does not affect the performance of Xpert MTB/RIF Assay. Fourthly, Xpert MTB/RIF Assay is very specific and is able to distinguish NTM from *Mtb*. Fifthly, Xpert MTB/RIF Assay was positive in seven patients who were sputum *Mtb* culture negative and were classified as having ‘Clinical TB’. Finally, the assay was easy to perform, gave results within two hours of sample processing, and there were no operational difficulties during its usage.

Our data validate and support the findings of a technical evaluation of the Xpert MTB/RIF Assay by Helb et al. [9] and a multicenter assessment at five trial sites in Peru, Azerbaijan, South Africa (Cape Town and Durban) and India by Boehme et al. [10]. While the sensitivity for smear positive samples was nearly 100% in all three studies, our analysis showed a substantially lower sensitivity in smear negative, culture positive samples than the two previous studies (71% Helb et al., 90% Boehme et al. and 61% in our study). Those differences might be explained by the following aspects: The previous studies included samples under much more selective conditions than our study which used samples from sequential patients with suspected TB without any pre-selection. Our study used concentrated sputum samples (which has been reported to increase sensitivity of smear microscopy by up to 39% [18]) and applied the revised WHO recommendation where one single scanty sputum (1–9/100 fields) defines a smear positive patient. The study from Boehme et al. [10] was performed on fresh sputum samples, while we used frozen samples. However, there is a controversy on whether the freezing process may cause some degradation of DNA or as discussed by Helb et al. [9] and Marlowe et al. [12] increases viscosity of the sample with improves recovery of mycobacterial nucleic acids.

TB-control, especially in TB/HIV-endemic areas with poor resources, is hampered by a lack of sensitivity and specificity of sputum smear microscopy which is often the only diagnostic method in place. In December 2010, WHO endorsed the Xpert MTB/RIF- Assay for widespread use. Among other indications the assay has a strong recommendation as initial diagnostic test in individuals with suspected HIV-associated TB [14]. HIV-infected patients are known to tend to have smear- negative sputum samples. This could be confirmed by our data showing that 88.9% of TB patients with a negative smear were HIV-infected. In this study we provide promise that the Xpert MTB/RIF Assay has a high diagnostic accuracy in HIV-positive patients (table 3) and that its performance is similar to that in HIV-negative patients in our cohort.

In terms of specificity, we found potentially false positive sputa from two patients detected by Xpert MTB/RIF Assay. One was a patient from group “No TB” (C−) whose samples were tested positive by Xpert MTB/RIF Assay. The fact that all three sputum samples obtained from that patient were consistently positive in the Xpert MTB/RIF Assay makes contamination an unlikely explanation. Furthermore, the patient had a history of previous TB, suggesting a sub clinical relapse or excretion of residual persistent DNA from dead organisms, as possible reasons for the positive result. The latter has been suggested to occur in treated TB patients [19]. The other false positive sputum sample was from a patient in the group “Clinical TB” (C−) who had NTM in sputum culture. *Mtb* was later also confirmed by additional Hain MTBDR*plus®* testing, which has a high specificity in clinical sputa [20], [21], and requires detection of *katG* and *inhA* amplification in addition to *rpoB* [*22*]. Therefore, non-specific amplification by the Xpert MTB/RIF Assay can be excluded. There was no history of previous TB, but the patient presented in an advanced stage of HIV immunosuppression (37 CD4^+^ T-cells/µl). It is possible that concomitant infection or colonization with *Mycobacterium fortuitum* which was detected in this patient's culture inhibited the slower growth of *Mtb* in culture, thereby preventing detection of the patient's TB by standard methods. If the reasoning in both these cases is assumed as correct, the specificity of Xpert MTB/RIF Assay would be 100% in our study.

Notwithstanding these exceptions, we are demonstrating here for the first time that Xpert MTB/RIF Assay can accurately differentiate between *Mtb* and NTM in clinical samples. This is relevant since NTM are commonly found in large geographical regions of Sub-Saharan Africa and are often picked up in sputum cultures, especially MGIT liquid cultures. Approximately one third of positive sputum cultures yield NTM in our setting in Mbeya, Tanzania. In our experience, these NTM are rather representing clinically insignificant concomitant flora of the sputum or saliva and are rarely causing disease in neither immunosuppressed nor immunocompetent individuals. Of 45 patients with NTM that were found in the groups of No TB (C−) and Clinical TB (C−), one patient tested positive by Xpert MTB/RIF Assay, resulting in a Xpert MTB/RIF Assay specificity of 97.8%. As described above there is good evidence to believe that this case was a true TB case. Recent analytical studies with NTM isolates by Helb et al. [9] and Blakemore et al. [23] indicated specificity of 100% for the Xpert MTB/RIF Assay, however, in a laboratory setting.

In our study Xpert MTB/RIF Assay detected seven TB cases (9,1%) in the group of patients with Clinical TB (C−) which were not picked up by any of the standard methods. Also, in the study by Boehme et al. [10] 29,3% of patients were classified as clinical TB cases who had no positive culture for *Mtb* but had a positive Xpert MTB/RIF Assay result. Unfortunately these cases were not delivered to final analysis. In our study these cases were followed over 56 days and we are confident based on the clinical evidence and the positive response to anti-TB treatment that they were true TB cases, even if the final bacteriological proof is missing.

Because of the difficulty that an imperfect reference standard poses when newer tests are evaluated that might be better than the reference standard, we suggest to introduce the term, “extended” reference standard to enable an alternative comparison of new and established assays. For our data the ‘extended’ reference standard would include as TB-positives those cases with a positive smear or culture and the seven Xpert MTB/RIF Assay positive cases with reasonable clinical evidence of active TB. If this definition was used the Xpert MTB/RIF Assay would have a higher or equal sensitivity in each per sample analysis than any of the standard methods alone or in combination. Thus sensitivity of Xpert MTB/RIF Assay would be 84.2% (95%CI = 74.0% to 91.6%) in one spot and 86.8% (95%CI = 77.1% to 93.5%) in one morning sputum compared to 84.2% (95%CI = 74.0% to 91.6%) and 79.0% (95%CI = 68.1% to 87.5%) respectively for all other methods combined (smear plus solid and liquid culture), (data not shown).

For future clinical practice, it is however of paramount importance to ascertain that these patients detected by Xpert MTB/RIF Assay only are unambiguously true TB cases that were missed by sputum culture and therefore a more thorough clinical evaluation study which specifically addresses these cases would be warranted. Furthermore, it will also be necessary to explore how this new, promising assay can be made accessible to developing countries. As with any new diagnostic test, the impact of Xpert MTB/RIF Assay will depend on the reproducibility of the results under actual field conditions, the manner and extent of their introduction, the strength of the laboratories and the degree to which access to appropriate therapy follows access to diagnosis. Our data suggest that especially smear-negative TB patients could benefit from the new assay in those areas where no culture is available. Both aspects, that analysis of only one single spot sputum sample by Xpert MTB/RIF-Assay can already reach reasonable sensitivity and that the result would be available on the day of sputum collection could result into more patients with active TB being diagnosed, avoiding loss of patients and treatment delay in those TB-suspects with a negative smear result who would undergo two ineffective empirical courses of antibiotics before TB-treatment would be initiated. Finally, this new sensitive and rapid diagnostic assay could lead to the reduction of the infectious pool and improvements in TB control.
